# Evaluating factors associated with the use of extracorporeal membrane oxygenation in major trauma – an analysis of the TraumaRegister DGU^®^

**DOI:** 10.1007/s00068-025-02844-4

**Published:** 2025-04-07

**Authors:** Maximilian Feth, Philipp M. Lepper, Christine Eimer, Andreas K. Bauer, Ralf Muellenbach, Jonas Ajouri, Matthias Ring, Gerhard Achatz, Jonathan Schober, Rolf Lefering, Bjoern Hossfeld, Martin Kulla

**Affiliations:** 1 Department of Anesthesiology, Critical Care, Emergency and Pain Medicine, German Armed Forces Hospital Ulm, Oberer Eselsberg 40, 89081 Ulm, Germany; 2 Department of Internal Medicine, Pulmonology and Critical Care, University Hospital of Bielefeld, Bielefeld, Germany; 3 Department of Anesthesiology and Critical Care, University Medical Center Schleswig-Holstein, Campus Kiel, Kiel, Germany; 4 Department of Anesthesiology and Critical Care, ECMO-Center, Klinikum Kassel, Kassel, Germany; 5 Department for Trauma Surgery and Orthopaedics, Reconstructive and Septic Surgery, Sportstraumatology, German Armed Forces Hospital Ulm, Ulm, Germany; 6 Institute for Research in Operative Medicine (IFOM), University Witten-Herdecke, Cologne, Germany

**Keywords:** ECMO, Respiratory failure, Major trauma, Circulatory failure, Extracorporeal life support

## Abstract

**Purpose:**

There is increasing evidence that use of ECMO is beneficial in major trauma patients with refractory organ failure. Hence, increased numbers of ECMO support following major trauma are reported. We set out to determine the use of ECMO among major trauma patients submitted to the TraumaRegister DGUr^®^ as well as patient features associated with ECMO support.

**Methods:**

The TraumaRegister DGU^®^ is a multinational database compiling trauma related health care data from point-of-injury, initial and critical care to outcome. Major trauma cases (AIS ≥ 3 irrespective of injury location) with subsequent critical care as well as respiratory and/or circulatory failure (SOFA score ≥ 3 per respective category) enrolled in the TraumaRegister DGU^®^ between 2015 and 2022 were reviewed. A logistic regression model was carried out to evaluate patient features associated with ECMO support.

**Results:**

410/ 22,548 individuals (1.8%) received ECMO support. Survival among ECMO patients was 46.1%. At discharge, good functional outcome as indicated by a Glasgow outcome scale > 3 was observed for 97 ECMO patients (23.6%). Age > 65 (OR 95%-CI 1.90, 1.52–2.60), male sex (OR 1.49, 95%-CI 1.41–1.95), coagulopathy at admission to the emergency department (OR 2.37, 95%-CI 1.88-3.00), chest trauma (OR 2.12, 95%-CI 1.61–2.81), sepsis (OR 2.94, 95%-CI 1.93–2.97), as well as massive transfusion (OR 2.23, 95%-CI1.56-3.19) were associated with the use of ECMO following trauma.

**Conclusion:**

In the TraumaRegister DGU^®^, ECMO for trauma related organ failure remains rare. Among ECMO patients, good functional outcome was observed infrequently. However, the design of the registry did not allow for capturing granular data on ECMO management and timing of organ failure. Hence, outcome data should be interpreted with caution. Nevertheless, evaluation of factors associated with ECMO support after trauma might contribute to early identification of ECMO candidates and improve patient distribution for trauma centers without ECMO capability.

## Background

Major trauma remains a leading cause of death worldwide [1]. While most potentially preventable deaths occur early after injury, organ failure contributes significantly to delayed death after trauma [2–4]. Major trauma leads to acute cardiopulmonary failure either directly via chest trauma or indirectly via sepsis or treatment associated, e.g., by fluid overload or massive transfusion [5, 6]. Although survival of patients suffering from acute respiratory distress syndrome (ARDS) after trauma has been reported superior to those suffering from non-traumatic ARDS, management of respiratory failure after trauma might be demanding [7, 8]. Since prone positioning might be contraindicated in trauma victims with severe traumatic brain injury (TBI) or spine trauma, advanced management of respiratory failure remains challenging.

Extracorporeal membrane oxygenation (ECMO) is a potentially lifesaving measure to address refractory respiratory or circulatory failure [9]. Although the first successful implementation of ECMO was performed in a trauma victim in 1971, the use of ECMO in trauma casualties has not been widespread over recent decades [10]. Particularly in patients with TBI, ECMO was often withheld since systemic anticoagulation is required [11, 12]. More recently, increasing rates of ECMO support in major trauma patients were reported due to technical improvements [13]. Positive outcomes from use of ECMO in major trauma were initially questioned, however, a recent meta-analysis compiling 1,822 major trauma patients with ECMO support emphasized a beneficial role of ECMO in trauma-associated refractory cardiopulmonary failure [14, 15]. In addition, timing of ECMO implementation appeared critical, hence, identification of patients requiring ECMO support after trauma was crucial for successful management [16]. Despite a few larger registry studies and meta-analyses, evidence for ECMO in major trauma patients primarily relies on case series and smaller cohort studies. Data on patient features associated with increased rates of ECMO use in trauma derived from comprehensively collected health data unfortunately is lacking.

According to the most recent research, ECMO after major trauma appears promising. However, frequency of ECMO use in countries contributing to the TR-DGU remains unclear. Hence, this analysis aims to (1) determine the frequency of ECMO use among patients with major trauma among cases submitted to the TR-DGU, (2) explore their outcome as well as identify factors associated with ECMO use after major trauma and, (3) evaluate demographic and clinical differences between ECMO survivors and non-survivors.

## Methods

### TraumaRegister DGU

Established in 1993, the TraumaRegister DGU^®^ (TR-DGU) is a multinational database compiling pseudonymized trauma related health data prospectively from the point of injury and prehospital care to the emergency room to include initial surgery and critical care management as well as discharge and outcome data. Additionally, demographics, injury patterns, comorbidities, point-of-care laboratory, and transfusion regimen are captured. General inclusion criteria for enrollment in the TR-DGU are (1) hospital referral with trauma team activation, (2) admission to the emergency room with vital signs or under ongoing chest compressions, (3) subsequent admission to critical or intermediate care units or, (4) death in the emergency or during initial surgery. Trauma centers contributing to the TR-DGU are mainly located in Germany (90%). However, a rising number of hospitals of other countries (e.g., Belgium, Finland, Luxembourg, the Netherlands, Austria, Switzerland, Slowenia, China or the United Arab Emirates) participate in the TR-DGU. For certified German trauma centers (local, regional, supraregional), contribution to the TR-DGU is mandatory. Therefore, a vast majority of German hospitals involved in trauma care participate in the TR-DGU. For hospitals located in countries other than Germany participation in the TR-DGU is voluntarily.

The Academy for Trauma Surgery (AUC), affiliated with the German Trauma Society (DGU), is responsible for documentation, data management and analysis, while the Committee on Emergency Medicine, Intensive Care and Trauma Management (Sektion NIS) provides scientific guidance. Data analysis from the TR-DGU requires clearance by the NIS following an internal peer review process. Using an online application, pseudonymized data is entered into the registry by participating hospitals. Data is captured via two different forms: a basic data form encompassing demographics, injury pattern and outcome necessary for quality assurance purposes and the full data set, which is usually submitted by larger trauma centers. Data collection forms can be assessed under https://www.auc-online.de/unsere-angebote/medizinische-register/traumaregister-dgu/.

This study obtained peer review clearance by the Sektion NIS (TR-DGU project ID 2022-017). Because data is collected and analyzed anonymously, the ethics committee of the University of Ulm waived IRB review (correspondence as of 06.01.2024).

### Study cohort

As the basic data set does not provide data on ECMO or detailed critical care management, only cases submitted with the standard data set were eligible for enrollment in the present study. As documentation of ECMO support within the TR-DGU started in 2015, we limited our analysis to cases documented between 2015 and 2022. Major trauma was defined by an Abbreviated Injury Score (AIS) ≥ 3 irrespective of injury location. Cases outside Europe as well as minor injury cases, defined by an AIS < 3, were excluded from the analysis [17]. ECMO support is predominantly used for refractory respiratory or circulatory failure, hence, cases without admission to the ICU, without mechanical ventilation, or without respiratory or circulatory failure were excluded from this study. Finally, cases without outcome information, e.g. due to transfer to another hospital after initial resuscitation, were excluded. Comparisons have been performed between cases with or without ECMO support as well as between ECMO survivors and non-survivors.

### Data

In this analysis, basic demographic data (e.g., age, sex) as well as the injury pattern, and outcome data were captured. To ensure a comprehensive description of major trauma within this analysis, fulfillment of the Berlin definition of polytrauma was evaluated in addition to the injury severity score and the AIS [18, 19]. Organ failure is defined by a sequential organ failure assessment (SOFA) score of at least two per organ category [20]. Body regions, for which an AIS of at least 3 was documented, were referred to as seriously injured. Multi organ failure was defined as dysfunction of at least 2 organ systems for at least 2 days. Coagulopathy at admission at the emergency department was defined as PTT ≥ 40 s., or Quick ≤ 60%, or INR ≥ 1.4. To predict the risk of death at hospital admission, the Revised Injury Severity Classification, version II (RISC II) was used [21]. The Glasgow Outcome Scale (GOS) was used to describe functional patient condition at discharge from the hospital [22].

### Statistics

Data analysis was performed using SPSS statistical software (SPSS Version 29, IBM Inc., Armonk, NY, USA). Data is presented as numbers and percentage for categorical data, and as mean with standard deviation (SD) for continuous data. In case of skewed data, median with quartiles was used instead. Observed differences were evaluated with Fisher’s Exact test, Mann-Whitney U-test, or t-test, as appropriate.

To identify factors independently associated with the use of ECMO in trauma patients, we performed a multivariate logistic regression analysis. For this model, the following predictors have been used: transfusion of packed red blood cells (pRBC), serious injury of head, chest, abdomen and extremity (AIS ≥ 3), male gender, age above 65 years, polytrauma according to the Berlin definition, and the presence of either sepsis or coagulopathy. Factors which have been included into the multivariate analysis were chosen following expert opinion and clinical reasoning. Results from the multivariate analysis are presented as odds ratios (OR) with 95% confidence intervals (CI). An OR below 1 indicates a lower probability for ECMO support, while an OR above 1 indicates an increased probability. Statistical significance was accepted at *p* ≤ 0.05. As no adjustment for multiple testing has been performed, results need to be interpreted with caution.

## Results

**Fig. 1:**
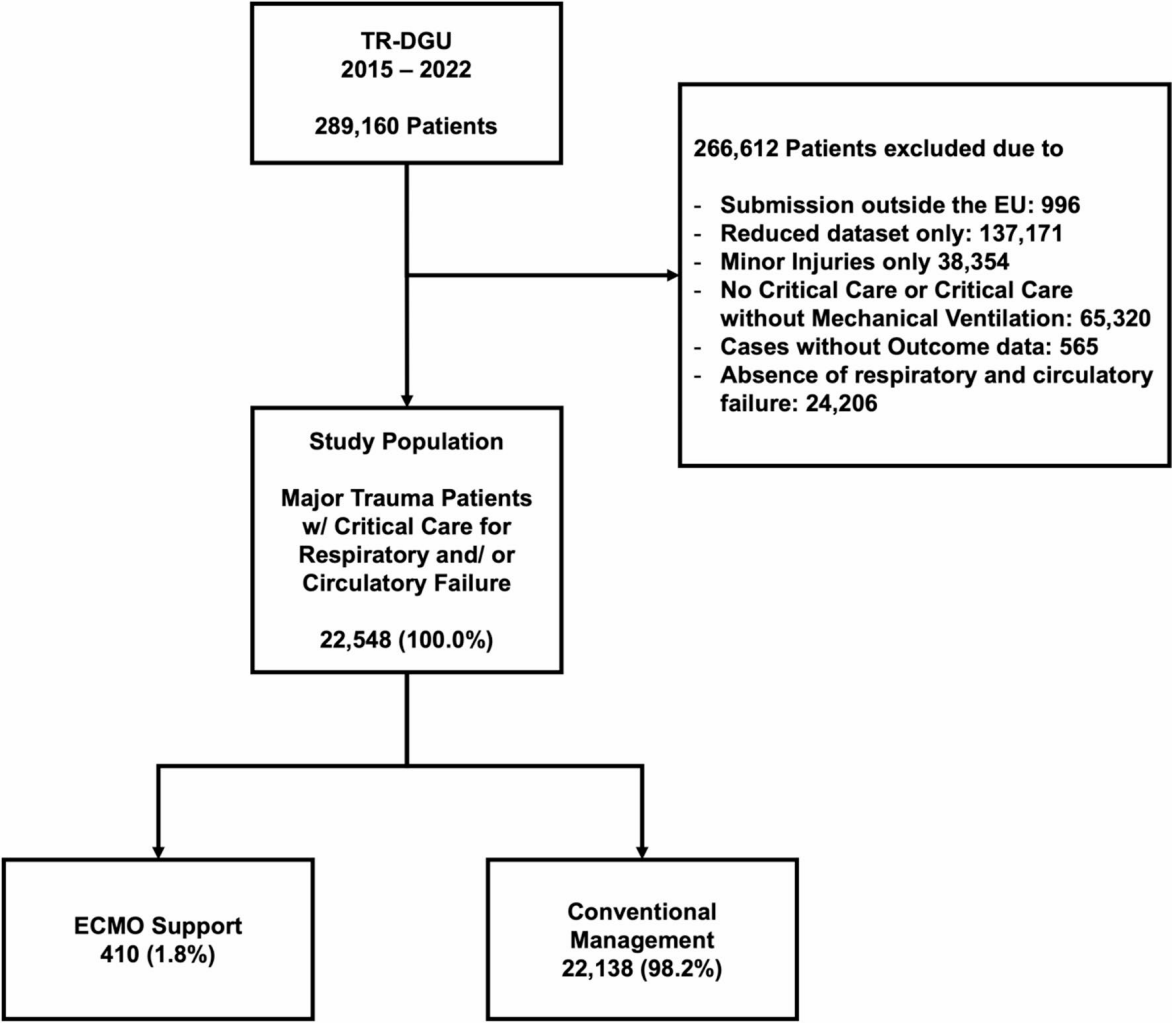
Study Cohort, Flowchart according to Study Criteria. Abbreviations *TR-DGU* TraumaRegister DGU^®^; *EU* European Union; *ICU* intensive care unit; *ECMO* extracorporeal membrane oxygenation

22,548 cases, including 410 individuals (1.8%) receiving ECMO support, met the inclusion criteria. (Fig. 1). Cases were predominantly submitted by German trauma centers (16,640; 2.2% ECMO) followed by Austrian (2,529; 0.9% ECMO) and Belgian facilities (1,622; 0.7% ECMO). Within the study period, annual trauma ECMO case numbers remained relatively stable between 1.4 (2020) to 2.1% (2016). Among the 251 trauma centers which submitted cases to the registry, 90 (35,9%) reported the use of ECMO. Of these 90, 12 trauma centers (13,2%) submitted at least 10 cases of ECMO support in major trauma during the study period. The majority of all ECMO cases (384, 93.7%) occurred at level I trauma centers.

### Outcome, clinical presentation, injury, and organ failure pattern

**Fig. 2:**
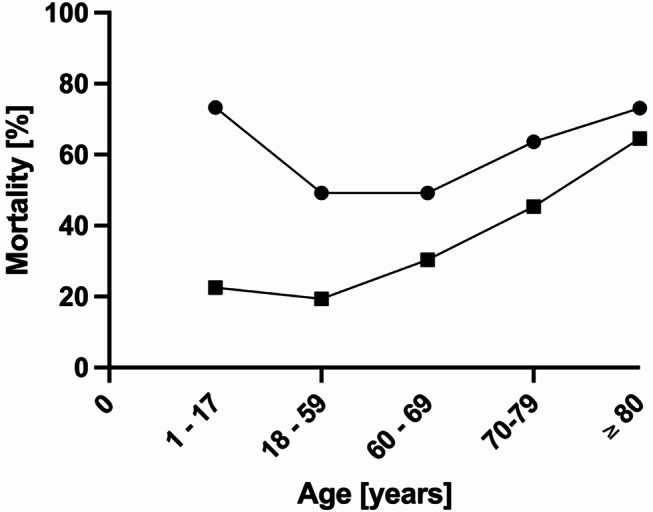
Mortality among Major Trauma Patients with (dot) or without ECMO support (box) categorized by age

For patients supported with ECMO, the leading cause of death was multiple organ failure (67.4%), followed by TBI (14.9%). Among cases without ECMO, most patients died due to TBI (54.6%) followed by multiple organ failure (31.3%). Both predicted (based on RISC-II, 32.9 vs. 26.8%, *p* < 0.001) and observed mortality (53.9% vs. 32.4%, *p* < 0.001) were higher among ECMO patients. Table 1 summarizes the distribution of ECMO use categorized by age, while Fig. 2 demonstrates survival of patients with and without ECMO support by age groups.

**Table 1:** Frequency of ECMO support among major trauma patients in different age groups

Age [years]	ECMO [%]	Conventional Management [%]
1–17	30 (3,5)	836 (96,5)
18–59	248 (2,2)	10,884 (97,8)
60–69	61(1,8)	3366 (98,8)
70–79	44 (1,2)	3618 (98,8)
≥ 80	26 (0,8)	3,421(99,2)

A combination of respiratory and circulatory failure was observed more often in ECMO patients (69.5% vs. 35.3%, p < 0.001). Respiratory failure in the absence of circulatory failure was documented for 13.9% ECMO cases and 13.7% cases without organ support. Mortality of ECMO patients was 22.8% for patients with isolated respiratory failure and 55.9% with isolated circulatory failure.

Patients with ECMO support experienced coagulopathy (48.8% vs. 25.4%, *p* < 0.001) and thrombembolic events (20.2% vs. 7.5%; *p* < 0.001) more frequently, while massive transfusion was also carried out more often (15.0% vs. 3,8%, *p* < 0.001). Table 2 summarizes a basic demographic description and clinical data while injury and organ failure patterns are presented in Table 3.

**Table 2:** Basic Demographics, Patient Distribution and General Clinical Management among major trauma patients with or without ECMO support

	Statistical Means	No ECMO n = 22,138	ECMO n = 410	p
Male	Count (%)	16,249 (73.4)	332 (81.0)	**< 0.001**
Age [years]	Mean ± SD	55.6 ± 21.6	47.7 ± 21.0	**< 0.001**
ASA > 2	Count (%)	5,360 (27.1)	81 (21.9)	**0.030**
Hospital Mortality	Count (%)	7174 (32.4)	221 (53.9)	**< 0.001**
Expected mortality rate based on RISC-II (%)	%	26.8	32.9	**< 0.001**
Level of Trauma Center	Count (%)			
- I		20,368 (92.0)	385 (93.9)	
- II		1,632 (7.4)	23 (5.6)	0.372
- III		138 (0.6)	2 (0.5)	
Primary Admission to Final Trauma Center	Count (%)	18,933 (85.5)	337 (82.2)	0.063
Hospital LoS [days]	Median (IQR)	19 (8–34)	17 (7–40)	0.725
ICU LoS [days]	Median (IQR)	12 (5–23)	15 (6–32)	**< 0.001**
Mechanical Ventilation [days]	Median (IQR)	6 (2–15)	10 (3–23)	**< 0.001**
Prehospital Fluid Resuscitation [ml]	Mean ± SD	811 ± 713	861 ± 695	0.086
Fluid Resuscitation in ED [ml]	Mean ± SD	2,049 ± 2,331	2,746 ± 2866	**< 0.001**
Transfusion of pRBC	Count (%)	5,823 (26.3)	181 (44.4)	**< 0.001**
Massive Transfusion	Count (%)	846 (3.8)	61 (15.0)	**< 0.001**
Coagulopathy	Count (%)	5,360 (25.4)	190 (48.8)	**< 0.001**
Thrombembolic Event	Count (%)	1.519 (7.5)	77 (20.2)	**< 0.001**

Abbreviations: *ASA* American Society of Anesthesiologists physical status; *ECMO* extracorporeal membrane oxygenation; *ED* emergency department; *ICU* intensive care unit; *IQR* interquartile Range; *LOS* length of stay; *pRBC* packed red blood cells, *SD* standard deviation

**Table 3:** Injury and organ failure pattern among major trauma patients with or without ECMO support

	Statistical Means	No ECMO *n* = 22,138	ECMO *n* = 410	*p*
Polytrauma (Berlin definition)	Count (%)	9,174 (41,4)	241 (58,8)	**< 0.001**
ISS	Mean ± SD	29.7 ± 13.1	36.4 ± 15.3	**< 0.001**
Blunt trauma	Count (%)	20,003 (95.6)	365 (94.8)	0.459
Relevant Injury per body region (AIS > 2)	Count (%)			
- Head		14,473 (65.4)	207 (50.5)	**< 0.001**
- Chest		11,718 (52.9)	315 (76.8)	**< 0.001**
- Abdomen		3,692 16.7)	131 (32.0)	**< 0.001**
- Extremity		6,906 (31.2)	173 (41.2)	**< 0.001**
Organ failure	Count (%)			
- Heart/circulation		19,113 (86.3)	353 (86.1)	0.891
- Respiratory		10,848 (49.0)	342 (83,4)	**< 0.001**
- Brain		12,565 (56,8)	234 (57.1)	0.919
- Renal		2,594 (11.7)	171 (41.7)	**< 0.001**
- Liver		1,039 (4.7)	101 (24.6)	**< 0.001**
- Coagulation		4,554 (20.6)	191 (46.6)	**< 0.001**
Sepsis	Count (%)	3,965 (18.9)	148 (37.3)	**< 0.001**
Multi Organ Failure	Count (%)	16,683 (75,4)	363 (88,5)	**< 0.001**

Abbreviations *AIS* abbreviated injury scale; *ECMO* extracorporeal membrane oxygenation; *ISS* injury severity score, *RISC* revised injury severity classification; SD standard deviation

Interestingly, we did not observe a difference in pre-trauma health condition as suggested by the ASA state (ASA > 2: 21.7% vs. 15.5%, *p* = 0.313) between ECMO survivors and non-survivors. Although not statistically significant, there was a tendency towards a lower injury severity for ECMO survivors (34.5% vs. Non-Survivors 38.0%, *p* = 0.051). Among survivors, acceptable outcome indicated by a Glasgow Outcome scale of at least 4 was observed for 97 cases (51.9%). Table 4 depicts further comparisons between ECMO survivors and non-survivors.

**Table 4:** Basic demographics, general clinical management, complications, injury, and organ failure in survivors and non-survivors undergoing ECMO (*n* = 410)

	Statistical Means	Survivor *N* = 189 (46.1%)	Non-survivor *N* = 221 (53.9%)	*p*
Male	Count (%)	151 (79.9)	181 (81.9)	0.616
Age [years]	Mean ± SD	46.3 ± 19.3	48.8 ± 22.3	0.262
ISS	Mean ± SD	34.5 ± 13.6	38.0 ± 16.5	0.051
Expected risk of death based on RISC II		21.2%	42.9%	**< 0.001**
ASA > 2	Count (%)	33 (17.5)	48 (21.7)	0.313
Discharge Mode	Count (%)			
- Home		35 (18.5)	n/a	n/a
- Rehabilitation		94 (49.7)		
- Other Hospital		50 (26.5)		
- Other		10 (5.3)		
Mechanical Ventilation [days]	Median (IQR)	19 (9–30)	5 (1–13)	**< 0.001**
ICU LOS [days]	Median (IQR)	29 (16–43)	7 (2–14)	**< 0.001**
Hospital LOS [days]	Median (IQR)	37 (24–54)	8 (2–15)	**< 0.001**
Massive Transfusion	Count (%)	20 (10.6)	41 (18.7)	**0.026**
Coagulopathy	Count (%)	66 (36.1)	124 (60.2)	**< 0.001**
Thrombembolic event	Count (%)	37 (21.4)	40 (19.1)	0.61
Organ failure	Count (%)			
- Circulation		145/189 (76.7)	208/221 (94.1)	**< 0.001**
- Respiration		159/189 (84.1)	183/ 221 (82.8)	0.790
- Brain		74/189 (39.2)	160/221 (72.4)	**< 0.001**
- Renal		58/189 (30.7)	113/221 (51.1)	**0.001**
- Liver		31/189 (16.4)	70/221 (51.1)	**< 0.001**
- Coagulation		66/189 (34.9)	125/221 (56.5)	**< 0.001**
Multi Organ Failure	Count (%)	149 (78.8)	214 (96.8)	**< 0.001**
Sepsis	Count (%)	71 (39.0)	77 (35.8)	0.533
Glasgow Outcome Scale	Count (%)			
- Neurovegetative state		10/189 (5.3)	n/a	n/a
- Severe Disability		80/189 (42.8)		
- Moderate Disability		60/189 (32.1)		
- Good recovery		37/189 (19.8)		
Relevant injury per body region (AIS > 2)	Count (%)			
- Head		84/18 (44.4)	123/221 (55.7)	**0.029**
- Chest		148/189 (78.3)	167/221 (75.6)	0.558
- Abdomen		51/189 (27.0)	80/221 (36.2)	0.056
- Extremity		87/189 (46.0)	86/221 (38.9)	0.161

Abbreviations *AIS* abbreviated injury scale; *ASA* American Society of Anesthesiologists physical status; *ECMO* extracorporeal membrane oxygenation; *ICU* intensive care unit; *IQR* interquartile range; *ED* emergency department; *LOS* length of stay; *SD* standard deviation

## Factors associated with ECMO support in major trauma

**Fig. 3:**
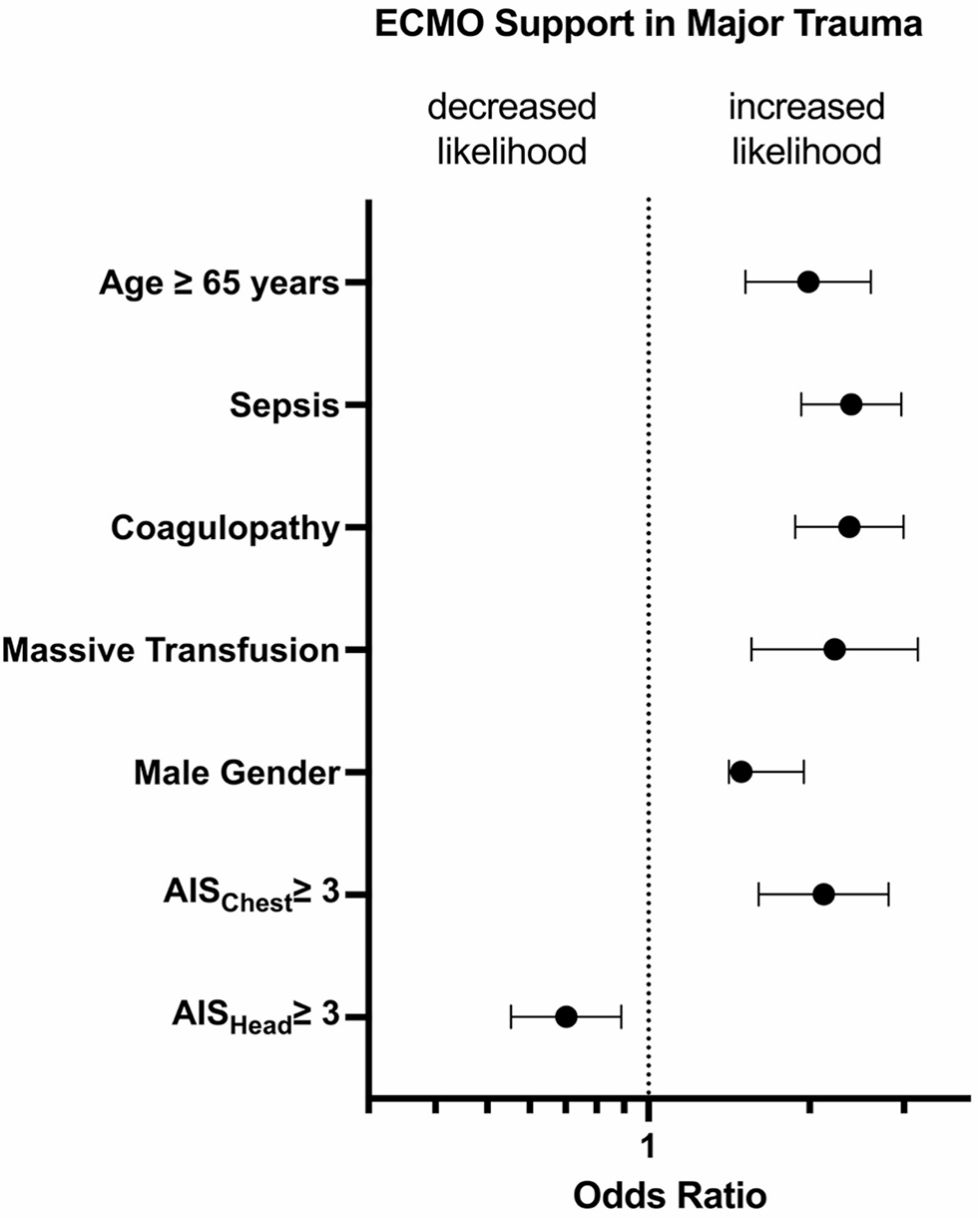
Patient features associated with the Likelihood of ECMO Support among Patients with Major Trauma (Forrest Plot). Data is presented as Odds Ratios and 95% confidence interval. Odds Ratios > 1 indicate an increased likelihood of ECMO support. Only independently associated patients’ features are displayed. Abbreviations *ECMO* extracorporeal membrane oxygenation; *AIS* abbreviated injury scale

A multivariate logistic regression model was carried out to identify patient specifics associated with the likelihood of ECMO support (Table 5; Fig. 3). While head injury was associated with a lower probability of ECMO support (OR 0.70; 0.55–0.89), age above 65 years (OR 1.99; 1.52–2.6) and male gender (OR 1.49; 1.14–1.95) were seen as factors associated with an increased probability for ECMO support. Additionally, ECMO support was associated with relevant chest trauma (OR 2.12; 1.61–2.81) and massive transfusion (2.23; 1.56–3.12) as well as the presence of sepsis (2.39; 1.93–2.97) or coagulopathy (2.37; 1.88–2.96). Serious injury of body regions other than head or chest, fulfilling the Berlin definition of polytrauma or pre-existing impaired health conditions as suggested by an ASA state above 2 did not significantly correlate with ECMO support within the study cohort. In contrast to an overall frequency of ECMO support of 1.8% within the study cohort, we observed frequencies from 0.7% for cases without any factor associated with ECMO support up to 13.8% for cases, which fulfilled 5 of the factors identified above. No case fulfilled all six features associated with an increased probability of ECMO support.

**Table 5:** Multivariate logistic regression analysis for the likelihood of ECMO support. (Nagelkerke’s R^2^ = 0.092)

	Coefficient	Odds Ratio [95% CI]	*p*
Injury Pattern (reference: AIS < 3)			
AIS_Head_ >= 3	-0.353	0.70 [0.55–0.89]	**0.003**
AIS_Chest_ >= 3	0.753	2.12 [1.61–2.81]	**< 0.001**
AIS_Abdomen_ >= 3	0.199	1.22 [0.95–1.57]	0.121
AIS_Extremity_ > = 3	-0.176	0.84 [0.66–1.07]	0.155
Sex (reference: female)			
Male	0.400	1.49 [1.41–1.95]	**0.004**
PRBC Transfusion until ICU Admission (reference: no transfusion)			
1–9 PRBCs	0.131	1.14 [0.88–1.47]	0.316
>= 10 PRBCs	0.801	2.23 [1.56–3.19]	**< 0.001**
Coagulation (reference: no coagulopathy)			
Coagulopathy	0.864	2.37 [1.88–3.00]	**< 0.001**
Polytrauma according to the Berlin definition (reference: no polytrauma)			
Polytrauma	0.013	1.01 [0.76–1.35]	0.927
Pre-existing Health Condition (reference: ASA 1 / 2)			
ASA 3 / 4	0.097	1.10 [0.83–1.46]	0.493
Inflammatory State (reference: no sepsis)			
Sepsis	0.873	2.94 [1.93–2.97]	**< 0.001**
Age (reference: age < 65 years)			
Age > = 65 years	0.688	1.90 [1.52–2.60]	**< 0.001**

Abbreviations *95-CI* 95% confidence interval; *AIS* abbreviated injury scale; *PRBC* packed red blood cells; *ASA* American Society of Anesthesiologists physical status

## Discussion

This analysis examines the use of ECMO in major trauma patients enrolled in the TR-DGU as well as factors associated with ECMO support after trauma. Within the major trauma patient cohort, 1.8% received ECMO resulting in a 46.1% survival and discharge rate. In this cohort, age above 65 years, male gender, massive transfusion, chest trauma, sepsis, and coagulopathy were independently associated with an increased likelihood of receiving ECMO support, while the presence of traumatic brain injuries prevented from ECMO support.

Given the typically younger age and lower comorbidity burden observed in trauma patients compared to those with non-traumatic ARDS, survival outcomes following ECMO support are generally considered more favorable in the trauma population. While recent literature cites survival to discharge rates between 65.9% and 69.1% for major trauma patients supported by ECMO, the cohort analyzed exhibited an overall survival to discharge of 46.1% [14, 23]. Unfortunately we cannot report granular data on ECMO initiation and management, making the comparison of our findings to those mentioned earlier challenging.

Specifically looking at age, it is noted that the mean age of our analysis was remarkebly higher than a meta-analysis conducted by Zhang and colleagues (48 years vs. 36 years) [14]. The higher mean age could be explained by the trend in Germany to provide invasive and specialized care for elderly and high-risk patients. This is consistent for the use of ECMO as well as mechanical ventilation, even for patients above 80 years [24, 25]. In our analysis, mortality in ECMO patients was increased in patients under 18 years and older than 69.

In Germany, mortality of veno-venous ECMO (VV-ECMO) patients decreased from 66 to 53% between 2010 and 2016, while the mortality of veno-arterial ECMO (VA-ECMO) patients increased from 58 to 66%. Although the overall mortality in this analysis is higher compared to international trauma ECMO data, mortality for major trauma patients with ECMO support irrespective of the choice of ECMO is lower than the overall mortality among patients treated with ECMO for non-traumatic organ failure in comparable German settings. As the elderly and high-risk patients are captured both in German health claim data and in our dataset, this comparison appears more reasonable. Moreover, functional outcome after ECMO support for trauma-related organ failure indicated by a GOS above 3 is still limited, yet promising refering to ECMO survivors only. Taken together, this emphasizes the potentially beneficial effect of ECMO in trauma related organ failure.

Survival for patients supported by VA-ECMO following trauma is lower compared to those supported by VV-ECMO [14]. Although we cannot report on the ECMO setup based on our data, we observed a hospital mortality of only 22.8% for ECMO patients with respiratory without circulatory failure and 55.9% for patients with circulatory but no respiratory failure. Assuming that VV-ECMO was primarily used for patients with respiratory, but not circulatory failure, ECMO for isolated respiratory failure after trauma might be beneficial.

For both ECMO and non-ECMO patients, the observed mortality was higher than the predicted mortality based on RISC-II – a score designed to predict survival after major trauma based on e.g., type of injury and initial presentation. Although the grade of injury as assessed by the RISC-II might correlate with organ injury, no definitive parameters of organ failure are included in this model. The study population, comprising patients exhibiting respiratory failure, circulatory failure, or a combination thereof, constituted a highly specific cohort, in which the predicted mortality based on RISC-II was likely to be underscored.

In 2022, Weidemann and colleagues demonstrated improved outcomes for patients with trauma-related ARDS when ECMO was started early [16]. Similar findings have been made for COVID-19- and Influenza-related ARDS as well as VA-ECMO related to cardiogenic shock [26–28]. With the number of trauma centers with adequate ECMO experience in Germany appearing limited, referral of patients with refractory organ failure to another trauma center may be required. To streamline patient distribution and resource allocation, timely identification of trauma patients with a potential need for ECMO support seems crucial. Evaluation of factors, which have been associated with ECMO use in this cohort, might contribute to a timely identification of such ECMO candidates, therefore contributing to a timely and physiology based decision process. Rigorous monitoring for early onset of respiratory or circulatory failure in conjunction with early consultation or even referral to a regional ECMO center should be considered.

As ECMO requires systemic anticoagulation, its use in patients with significant TBI was often withheld. However, there is evolving evidence for the use of ECMO after TBI. In 2023, Hatfield and colleagues examined outcomes of 108 patients supported by ECMO after trauma which resulted in a hospital mortality rate of 33.9% [29]. They also identified younger age and male gender as risk factors linked to the requirement of ECMO after trauma. However, in Hatfield’s study, patients undergoing ECMO presented with a reduced trauma load and were generally younger (30 vs. 48 years), potentially contributing to improved survival rates. Consistent Hatfield’s findings, Mader et al. recently provided insights into the use of ECMO in TBI based on the TR-DGU [30]. While this study primarily examines patients which overlap with the present investigation, Mader et al. reported a 51% hospital mortality. Among the survivors, 48% were discharged with a GOS > 3. Concurring with Hatfield, Mader and colleagues, utilization of ECMO might be beneficial for organ failure after trauma after careful consideration of inherent risks against potential benefits. Our cohort, in contrast, associated TBI with a decreased probability of ECMO after trauma. With additional research, Mader’s and Hatfield’s results may contribute to more frequent use of ECMO for TBI patients with refractory organ failure, especially as focused anticoagulation while a patient is on ECMO contributes to risk reduction.

ECMO is associated with both bleeding and thrombembolic complications. Among TR-DGU cases, we observed higher rates of thrombembolic events among ECMO patients, however, there was no difference between ECMO survivors and non-survivors. In a cohort of COVID-19 patients supported with ECMO, an increased risk for ICU mortality has only been seen for cases with bleeding, but not for those with thrombembolic complications [31]. Since, timing of any thrombembolic complication in relation to ECMO therapy is not captured in the TR-DGU, we can only speculate about the role of ECMO contributing to higher frequencies of thromboembolic events in organ support patients as well as its impact on mortality. Interestingly, neither thrombembolic nor bleeding complications ranked among the leading causes of death in either group in our analysis.

### Limitations

A major limitation of this analysis is the lack of data on ECMO setup and performance. The TR-DGU was initially established to guarantee benchmarking in trauma care as well as to facilitate nationwide trauma research throughout the continuum of trauma care. Despite duration of mechanical ventilation and critical care management, general transfusion and fluid regimen, detailed insights into critical care and ECMO procedures (e.g., configuration of ECMO, duration of ECMO, anticoagulation, ECMO-associated complications) are not provided in the data set. While thrombembolic complications are mentioned, it remains unclear, if they occurred during ECMO support or independently. Additionally, the study lacked granular detail regarding the onset of organ failure. Consequently, comparing groups with either isolated respiratory or isolated circulatory failure or combined organ failure was not reasonably possible. However, this study compiles a large set of trauma patients with comprehensively collected health care data. As most of the ECMO cases were submitted by a minority of participating trauma centers, we did not adjust for a potential center effect. Additionally, we did not test for or exclude factors from the multivariate analysis due to collinearity, although some factors such as age and pre-injury health condition might be associated. While detailed conclusions on ECMO management in trauma such as anticoagulation regimen or the ideal start of ECMO cannot be drawn from our study, this analysis allows demographic insights and presents factors associated with the use of ECMO in trauma. However, to further evaluate the effect of ECMO on both survival and functional outcome in major trauma patients, larger randomized controlled trials would be favourable.

## Conclusion

ECMO support in major trauma remains infrequent. Although recent evidence does not suggest TBI as a contraindication for ECMO, the presence of relevant TBI was associated with a reduced likelihood of ECMO support. Age above 65, male gender, presence of sepsis or coagulopathy and a relevant chest injury increased the likelihood of ECMO support. Among patients supported by ECMO, survival with good functional outcome appeared limited. However, the design of the registry did not allow for capturing granular data on ECMO management and timing of organ failure. Hence, outcome data should be interpreted with caution. Further research is needed to delineate factors associated with favorable functional outcome following ECMO support in major trauma.

## Data Availability

Data is available upon request by the authors.
